# The role of intrathecal free light chains kappa for the detection of autoimmune encephalitis in subacute onset neuropsychiatric syndromes

**DOI:** 10.1038/s41598-023-44427-6

**Published:** 2023-10-11

**Authors:** Dominic Bertram, Thanos Tsaktanis, Achim Berthele, Thomas Korn

**Affiliations:** 1 Department of Neurology, Technical University of Munich School of Medicine, Ismaninger Str. 22, 81675 Munich, Germany; 2 Department of Neurology, University Hospital Erlangen, Friedrich-Alexander University Erlangen-Nürnberg, Erlangen, Germany; 3 Institute for Experimental Neuroimmunology, Technical University of Munich School of Medicine, Ismaninger Str. 22, 81675 Munich, Germany; 4 Munich Cluster for Systems Neurology (SyNergy), Feodor-Lynen-Str. 17, 81377 Munich, Germany

**Keywords:** Neuroimmunology, Diagnostic markers

## Abstract

Intrathecal synthesis of free light chains kappa (FLCK) is increasingly recognized as a marker of inflammatory CNS pathologies. Here, we tested the performance of FLCK in differentiating autoimmune encephalitis (AIE) from non-inflammatory etiologies in subacute onset neuropsychiatric syndromes. Patients undergoing diagnostic work-up for suspected autoimmune encephalitis at our department between 2015 and 2020 were retrospectively assessed for definitive diagnosis, available CSF and blood samples, as well as complete clinical records. Intrathecal FLCK was measured along with established CSF markers of CNS inflammation. The study cohort consisted of 19 patients with antibody-mediated AIE (AIE^+^), 18 patients with suspected AIE but without detectable autoantibodies (AIE^–^), 10 patients with infectious (viral) encephalitis (INE), and 15 patients with degenerative encephalopathies (DGE). 25 age- and sex-matched patients with non-inflammatory neurological diseases (NIND) were used as a control group. All AIE^+^ patients exhibited intrathecal synthesis of FLCK compared to only 39% of AIE^–^ patients and 81% of patients in the INE group. No intrathecal synthesis of FLCK was found in DGE and NIND patients. While intrathecal FLCK was equally specific for an inflammatory etiology as oligoclonal bands (OCB) in the cerebrospinal fluid (CSF), the sensitivity of intrathecal FLCK for any inflammatory intrathecal process was higher than that of OCB (83% vs. 38%). Intrathecal FLCK synthesis was found to discriminate AIE^+^ from non-inflammatory encephalopathies and AIE^–^ when the CSF cell count was normal [receiver operating characteristic (ROC) analysis area under the curve (AUC): 0.867, p = 0.002], while it failed to differentiate between AIE^+^ and INE in the presence of CSF pleocytosis (AUC: 0.561, p = 0.607). In conclusion, in the absence of CSF pleocytosis, intrathecal FLCK discriminated AIE^+^ from competing diagnoses in our cohort of subacute onset neuropsychiatric syndromes. In addition to established markers of CSF inflammation, intrathecal FLCK might support clinical decision-making and contribute to selecting patients for (repeated) antibody testing.

## Introduction

Neuropsychiatric symptoms are among the most common reasons for neurologic consultation. Among many underlying disorders, encephalitis—if recognized timely—represents a treatable diagnosis^1^. Infectious causes of encephalitis tend to be reliably detected, while the diagnosis of autoimmune encephalitis (AIE) can be challenging^2^. The published diagnostic criteria have facilitated clinical decision-making and complemented the gold standard of antibody detection^3^. However, antibody test results return delayed (or negative) in an often dramatic clinical setting^4^. Conversely, studies and real-world evidence found that timely diagnosis and initiation of immunosuppressive therapy foster recovery and long-term independence in AIE patients^1,5^. While standard cerebrospinal fluid (CSF) parameters and polymerase chain reaction often allow a swift identification of infectious etiologies, the need for biomarkers to disentangle subtypes of AIE and guide management in the early phase of the diagnostic process has not been met^6^.

In addition to intact immunoglobulins, plasma cells in the systemic and CSF compartment produce excess monomeric free light chains (FLC) of either kappa (FLCK) or lambda (FLCL) subtype^7^. Intrathecal synthesis of FLCK is increasingly recognized as a marker of inflammatory CNS pathologies such as multiple sclerosis^8–11^. Like immunoglobulins, FLCK can be measured in CSF and serum by nephelometry. Standardized, commercially available nephelometric assays provide a fast, easy-to-analyze, and quantitative measure of intrathecal FLCK, which is relatively resilient to blood contamination, storage time, temperature, as well as some types of immunomodulation^12,13^. While stand-alone serum measures of FLCK do not faithfully reflect inflammation in the intrathecal space due to the blood-CSF barrier and various confounding factors in the systemic compartment, pairwise measures of FLCK in serum and CSF are used to calculate intrathecal synthesis, taking into account the blood-CSF barrier function. The Reiber diagram for the determination of the intrathecal fraction of FLCK (IF-FLCK) has been widely accepted in German-speaking countries and has gained some attention throughout Europe as it considers blood-CSF barrier function and diffusion of serum proteins. Therefore, it provides superior diagnostic accuracy as compared to linear ratio calculations of intrathecal FLCK^8,9,11,14,15^.

Here, we tested whether intrathecal FLCK synthesis (as defined by the Reiber diagram) can aid in discriminating AIE from relevant differential diagnoses in patients with subacute neuropsychiatric syndromes.

## Results

In neuropsychiatric syndromes with subacute onset, a diverse spectrum of differential diagnoses must be considered. CSF antibody testing (as the gold standard) for the diagnosis of AIE returns with a latency that may delay treatment and thus negatively affect the neurological outcome. In a cohort of suspected AIE patients, we tested whether FLCK could be an early marker of AIE to narrow the gap for timely intervention.

### Patient characteristics

**Figure 1:**
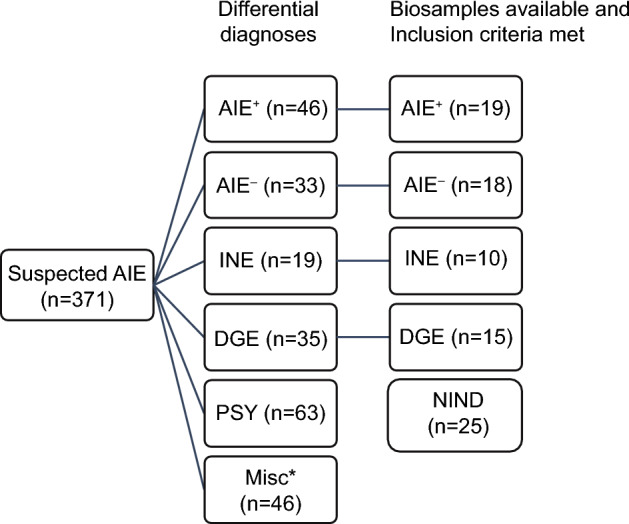
Graphical representation of the study cohort. Between 2015 and 2020, 371 patients underwent diagnostic work-up for suspected AIE. Diagnostic categories were AIE^+^ (antibody-positive autoimmune encephalitis), AIE^–^ (antibody-negative suspected autoimmune encephalitis), INE (infectious encephalitis), DGE (degenerative encephalopathy), PSY (psychiatric diagnoses). A large number of cases (n = 175) could not be assigned to any of the categories. After the application of exclusion criteria (see “Methods”) and screening for complete (blood and CSF) biosamples, 62 patients were included in the further analysis [87 individuals, including the control group of 25 patients with NIND (non-inflammatory neurologic disease)].

We screened 371 patients undergoing diagnostic work-up for suspected AIE between 2015 and 2020. One of the known anti-neural (anti-neuronal plus anti-glial) antibodies or antibodies against intracellular antigens was detected in 46 patients (12%). Thirty-three patients (9%) had a syndrome suggestive of AIE but no detectable antibody. Nineteen patients (5%) had encephalitis due to an infectious cause. Thirty-five patients (9%) were diagnosed with a degenerative disorder accounting for their encephalopathic syndrome, and 63 patients (17%) received autochthonous psychiatric diagnoses. The remainder of the syndromes (47%) were assigned to metabolic, neoplastic, toxic, or genetic etiologies, delirium, and unknown or pending causes at the time of the study (Fig. 1).

All study participants were assigned to distinct diagnostic categories. Patients with autochthonous psychiatric diagnoses were excluded since first episode psychosis without "red flags" (neurologic features, neurologic or malignant comorbidities, resistance or adverse effects of antipsychotics) allows for psychiatric diagnoses to be made with a reasonable degree of accuracy^16^. The exclusion criteria led to the drop-out of the following individuals: 71 patients were excluded due to incomplete clinical data or specimens, 63 patients due to autochthonous psychiatric diagnoses, and 175 patients due to miscellaneous, uncertain, or unknown diagnoses. Three patients were excluded from the statistical evaluation due to treatment with immunosuppressants (i.e. corticosteroids, rituximab, or azathioprine). Three patients were excluded owing to competing inflammatory diseases (Multiple sclerosis, CLIPPERS, Sjogren syndrome) and to a syndrome incompatible with AIE. One patient in the INE group was excluded due to sterile CSF. Eventually, 62 patients remained in the cohort and were stratified according to their diagnosis into AIE^+^, AIE^–^, INE, and DGE (Table 1). Details on the clinical characteristics of the AIE^+^ and AIE^–^ groups are listed in Supplementary Files 1 and 2.

**Table 1:** Patient characteristics.

	AIE^+^	AIE^–^	INE	DGE	NIND	p value
n	19	18	10	15	25	
Age, years	54 (19)	58 (21)	60 (15)	68 (8)	54 (22)	0.4311
Female/male, n	10/9	10/8	6/5	7/8	10/15	
Immunotherapy at sampling, n	0	0	0	0	0	
Q_Alb_ (10^-3^)	10.3 (6.5)	9.8 (6.4)	12.1 (7.3)	7.7 (4.2)	6.3 (2.4)	0.0468
Intrathecal Ig synthesis, n	3	2	4	0	0	> 0.05
Oligoclonal bands, n	6	6	5	0	0	
CC, /µL	10 (12)	57 (160)	104 (140)	2 (2)	1 (1)	< 0.0001
FLCK serum, mg/L	16.2 (10.4)	17 (12.3)	13.7 (6.7)	17.7 (6.4)	12.5 (3.4)	0.1425
FLCK CSF, mg/L	2.8 (6.2)	1.8 (3.9)	3.6 (5.8)	0.2 (0.2)	0.2 (0.1)	< 0.0001
FLCK index	15.3 (21.7)	8.9 (12.7)	24.7 (44.1)	1.8 (0.6)	2.1 (0.5)	< 0.0001
FLCK IF, % to Q_mean_	56.9 (33.5)	37.9 (42.3)	61.1 (32.5)	5.7 (10.4)	8.4 (8.8)	< 0.0001
FLCK IF, % to Q_lim_	40 (39.4)	31 (40.5)	44.2 (40.1)	0 (0)	0 (0)	< 0.0001
eGFR (CKD-EPI), mL/min/1.73 m^2^	90 (24)	86 (20)	95 (16)	81 (15)	86 (19)	0.7403
FACS available n (%)	19 (100)	15 (83)	10 (100)	11 (73)	6 (24)	
CD19^+^ B cells (%)	4.6 (4.9)	3.2 (3.1)	1.4 (1.4)	1.8 (3.3)	0.5 (0.3)	0.0006
CD19^+^CD138^+^ plasma blasts (%)	1.0 (1.6)	0.5 (1.2)	0.4 (0.7)	0.04 (0.06)	0.02 (0.06)	0.0028
CD4^+^ T cells (%)	62.7 (12.1)	63.7 (12.6)	62.1 (12.3)	66.6 (6.7)	61.7 (12.4)	0.9743
CD8^+^ T cells (%)	20.8 (8.9)	19.6 (7.2)	25.2 (15.1)	23.3 (4.3)	20.2 (5.4)	0.5326
CD14^+^ Monocytes (%)	2.6 (2.8)	3.1 (5.3)	2.0 (2.6)	5.0 (4.1)	6.9 (4.1)	0.0061
CD56^+^ NK cells (%)	3.3 (2.8)	3.9 (3.1)	4.9 (3.3)	2.6 (1.9)	2.4 (1.2)	0.339

Shared criterium (except NIND): Encephalopathic syndrome suggestive or compatible with autoimmune encephalitis. AIE^+^ (autoantibody-positive autoimmune encephalitis; total n = 19: NMDAR n = 6, LGI1 n = 4, CASPR2 n = 1, IgLON5 n = 1, GAD65 n = 1, GABA(a) n = 1, DPPX n = 1, PNMA2 n = 1, Anti-Yo n = 1, AGNA n = 1, VGCC n = 1), AIE^–^ (suspected autoimmune cause, no autoantibody in blood and CSF; total n = 18). INE (infectious encephalitis, no autoantibody in blood and CSF; total n = 10; HSV n = 4, FSME n = 4, JC-Virus n = 2). DGE (Degenerative encephalopathy, no autoantibody in blood and CSF; total n = 15; Creutzfeld–Jacob Disease n = 3, dementia n = 3, cerebral amyloid angiopathy n = 3, MSA-C n = 3, FTD n = 1, ALS n = 1, PSP n = 1). NIND (non-inflammatory neurological disease; total n = 25; headache n = 8, benign intracranial hypertension n = 8, normal pressure hydrocephalus n = 8, cranial nerve palsy n = 3). Values are depicted as mean (standard deviation) unless otherwise indicated.

A control group of 25 patients with non-inflammatory neurological diseases (NIND; headache, benign intracranial hypertension, normal pressure hydrocephalus, and cranial nerve palsy) was established, leading to a total number of 87 patients that were further analyzed.

### CSF standard parameters and renal function

CSF pleocytosis was found in 53% of the AIE^+^ group (n = 10/19), 44% of the AIE^–^ group (n = 8/18), and 90% of the INE group (n = 9/10). CSF pleocytosis was absent in the DGE and NIND groups. CSF cell counts differed significantly between the groups, with the highest white cell counts in the INE group [cells/µL, mean (SD): 104 (140)], intermediate counts in the AIE^–^ group [cells/µL, mean (SD): 57 (160)], and lowest counts in the AIE^+^ group [cells/µL, mean (SD): 10 (12)].

Blood-CSF barrier dysfunction (corrected for age) was most frequent in INE [n = 6/10 (60%)], followed by AIE^+^ [n = 8/19 (42%)] and AIE^–^ [n = 4/18 (22%) while least frequent in DGE (n = 3/15 (20%)] and NIND [n = 3/25 (12%)]. Intrathecal synthesis of either IgG, IgA, or IgM was present in 40% of INE (n = 4/10), 16% of AIE^+^ (n = 3/19), 11% of AIE^–^ (n = 2/18), and in none of either the DGE or NIND patients.

The presence of CSF-specific OCB was determined in all patients for whom paired CSF/serum samples were still available (n = 84/87). We found CSF-specific OCB in 32% of AIE^+^ patients (n = 6/19), in 33% of AIE^–^ patients (n = 6/18), in 50% of INE patients (n = 5/10), and in none of the individuals in the DGE and NIND groups.

Renal function was not found to be divergent across groups (Table 1). Overall, few patients (5/87) had substantially impaired renal function (here defined as eGFR < 60 mL/min/1.73 m^2^) at the time of sample acquisition: two patients in the AIE^+^ group (one with IF-FLCK 80% > Q_lim_, one with IF-FLCK < Q_lim_), two patients in the AIE^–^ group (both with IF-FLCK < Q_lim_), one patient in the NIND group (IF-FLCK < Q_lim_), and none in the INE and DGE groups.

### Immune cell subsets

**Figure 2:**
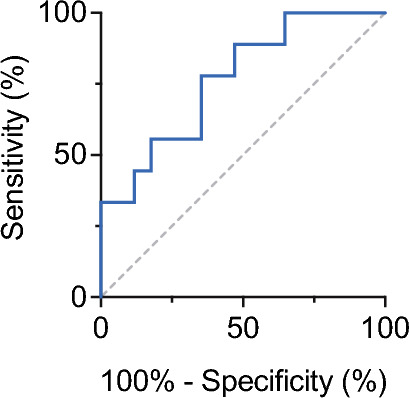
Receiver operating characteristics for the CSF fraction of B cells in the differentiation of AIE^+^ and INE under conditions of CSF pleocytosis and intrathecal FLCK synthesis > Q_lim_. AUC: 0.765, Cutoff 3.5%, p = 0.02.

The fractions of CSF immune cell subsets showed group-specific features, with few statistically significant intergroup differences (Supplementary File 3). CSF CD19^+^ B cell fractions were higher in the inflammatory group as compared to the non-inflammatory groups (p ≤ 0.001). We found the highest CSF B cell fractions in the AIE^+^ group [mean (SD): 4.6% (4.9)] and the AIE^–^ group [mean (SD): 3.2% (3.1)] and the lowest fraction in the INE group [mean (SD): 1.4% (1.4)]. Here, the group difference between AIE^+^ and INE reached statistical significance, providing a potential means to distinguish early infectious (viral) from autoimmune encephalitis when the remainder of the CSF parameters were consistent with an inflammatory etiology (Fig. 2).

### Intrathecal FLCK synthesis

**Figure 3:**
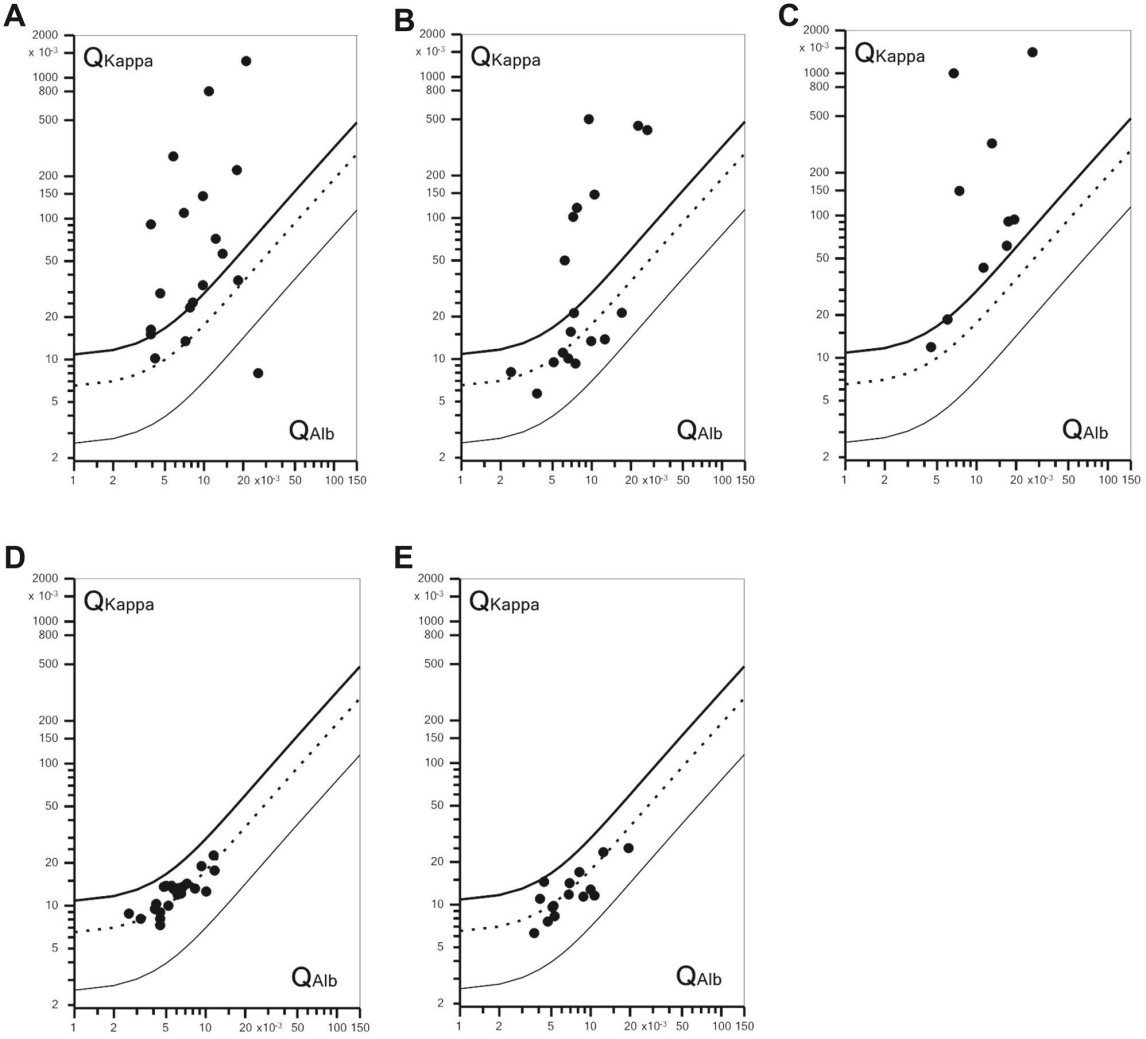
Double logarithmic quotient-diagrams (Reiber diagrams) of Q_FLCK_ (Q_Kappa_), i.e. FLCK CSF/serum ratio, set against Q_Alb_, i.e. Albumin CSF/serum ratio. (**A**) AIE^+^, 78% (n = 15/19) of Q_Kappa_ > Q_lim_; (**B**) AIE^–^, 39% (n = 7/18) of Q_Kappa_ > Q_lim_; (**C**) INE, 81% (n = 9/11) of Q_Kappa_ > Q_lim_; (**D**) DGE and (**E**) NIND, all Q_Kappa_ < Q_lim_. Symbols above Q_lim_ indicate intrathecal FLCK synthesis. *Q*_*lim*_ thick upper line, *Q*_*mean*_ dotted middle line, *Q*_*low*_ thin lower line.

When we assessed the CSF/serum ratio of FLCK, 78% (n = 15/19) of FLCK ratios in the AIE^+^ group fell above the upper discrimination line (Q_lim_), indicating intrathecal synthesis of FLCK (Fig. 3A, Supplementary File 1). Except for four patients with paraneoplastic syndromes (anti-Yo, anti-Ma2, anti-SOX1, and anti-VGCC), all AIE^+^ patients showed intrathecal synthesis of FLCK (n = 15/19). In contrast, only 39% of the individuals in the AIE^–^ group (n = 7/18) showed intrathecal FLCK synthesis (Fig. 3B, Supplementary File 2). In the INE group, 90% (n = 9/10) of FLCK ratios exceeded Q_lim_ (Fig. 3C). Only one patient with INE (JC-Virus infection) had an FLCK quotient below Q_lim_. None of the patients in the DGE and NIND groups exhibited intrathecal synthesis of FLCK (Fig. 3D,E).

For the relative amounts of intrathecally synthesized FLCK (as depicted by the IF-FLCK with reference to Q_mean_), we observed significant differences in intergroup comparisons between inflammatory (AIE^+^, AIE^–^, INE) and non-inflammatory (DGE, NIND) conditions (mean IF-FLCK 52.0% vs. 7.05%, p < 0.0001, Supplementary File 4). Accordingly, in ROC analyses, IF-FLCK showed similar performance in discriminating between inflammatory and non-inflammatory etiologies as the CSF white cell count (CSF cell count: AUC = 0.852, p = 0.0002; IF-FLCK: AUC = 0.809, p = 0.001, Supplementary File 4). No significant group differences in mean intrathecal FLCK fractions were found among the inflammatory groups AIE^+^, AIE^–^, and INE.

**Figure 4:**
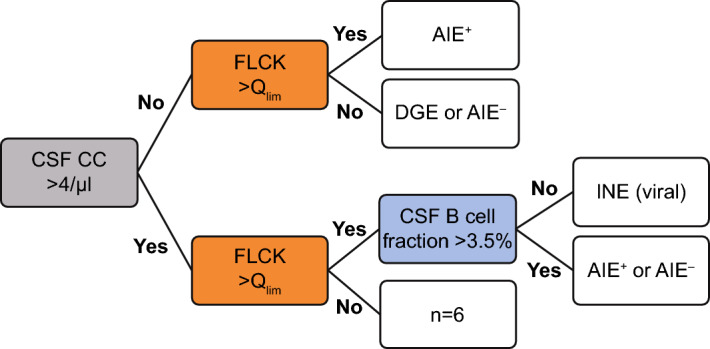
Sequential diagnostic approach applicable to this cohort using CSF white cell count, intrathecal FLCK synthesis, and CSF B cell fraction. Pre-stratification of the cohort into CSF pleocytosis (CC > 4/μL) and normocytosis. In the upper arm (CSF normocytosis), intrathecal FLCK synthesis discriminated AIE^+^ from other diagnoses. In the lower arm (CSF pleocytosis), Q_FLCK_ > Q_lim_ indicated either infectious or autoimmune encephalitis. In the event of CSF pleocytosis and Q_FLCK_ > Q_lim_, the intrathecal B cell fraction (< 3.5%) distinguished infectious from autoimmune encephalitis with AUC of 0.75.

**Figure 5:**
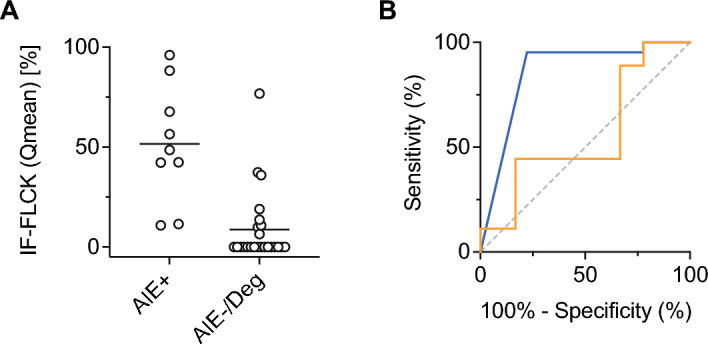
Test performance of IF-FLCK. (**A**) Individual IF-FLCK values for the CSF-normocytic subgroup (AIE^+^, AIE^–^, and DGE, for visualization displayed with reference to Q_mean_), (**B**) receiver operating characteristics with respect to Q_FLCK_ > Q_lim_ after stratification of the cohort into CSF pleocytosis and CSF normocytosis. In CSF normocytosis, the intrathecal fraction of FLCK (IF-FLCK) distinguished AIE^+^ from the rest (DGE, AIE^–^) with a sensitivity of 95.2% and a specificity of 77.8% (AUC 0.867, p = 0.002, blue line). In CSF pleocytosis, the capacity of IF-FLCK to discriminate AIE^+^ or AIE^–^ from INE was poor (AUC 0.561, p = 0.607, orange line).

After dichotomization of the cohort into one arm with CSF normocytosis (CC < 5/µL; comprising patients from the groups AIE^+^, AIE^–^, and DGE) and one arm with CSF pleocytosis (CC > 4/µL; comprising patients from the AIE^+^, AIE^–^, and INE groups, Fig. 4), we found the intrathecal fraction of FLCK to be significantly higher in AIE^+^ compared to AIE^–^ and DGE in the arm with CSF normocytosis (Fig. 5A). ROC analysis in this subset of patients showed IF-FLCK to differentiate AIE^+^ from AIE^–^ and DGE with high sensitivity and specificity (AUC = 0.867, p = 0.002, Fig. 5B). In the CSF pleocytosis arm, we found no significant differences in intrathecal FLCK fractions between the AIE^+^, AIE^–^, and INE groups.

Taken together, FLCK was more sensitive than CSF pleocytosis or CSF OCB to detect an inflammatory etiology (infectious *or* autoimmune) in this cohort of newly onset neuropsychiatric syndromes (Table 2). Intrathecal FLCK synthesis did not have any additional discriminative power to separate AIE from INE in case of an inflammatory CSF transformation. However, when the CSF cell count was normal, intrathecal FLCK synthesis in the earliest available CSF/serum samples was still indicative of an inflammatory etiology (e.g. antibody-positive AIE). Therefore, even though the markers of intrathecal inflammation investigated in this study (including intrathecal FLCK synthesis) widely overlapped in their diagnostic potency for an inflammatory process in the CSF, positive intrathecal FLCK synthesis should raise suspicion for an inflammatory etiology when routine CSF parameters (CSF pleocytosis, OCB and intrathecal Ig synthesis) are negative.

**Table 2:** Sensitivity and specificity of intrathecal FLCK synthesis (Q_FLCK_ > Q_lim_), OCB, and CSF white cell count (CSF-CC) in inflammatory vs. non-inflammatory neurologic diseases in our cohort.

	Inflammatory neurologic disease n = 29	Non-inflammatory neurologic disease n = 40	Total n = 69
Q_FLCK_ > Q_lim_	n = 24	n = 0	PPV 1.0
OCB positive	n = 11	n = 0	PPV 1.0
CSF-CC > 4/µL	n = 19	n = 0	PPV 1.0
Q_FLCK_ < Q_lim_	n = 5	n = 40	NPV 0.89
OCB negative	n = 18	n = 36	NPV 0.67
CSF-CC < 5/µL	n = 10	n = 40	NPV 0.8
	Sensitivity FLCK 83% Sensitivity OCB 38% Sensitivity CSF CC 66%	Specificity FLCK 100% Specificity OCB 100% Specificity CSF CC 100%	

Inflammatory neurologic disease = AIE + and INE (AIE^–^ was excluded from this analysis due to diagnostic uncertainty); non-inflammatory neurologic diseases = DGE and NIND. PPV, positive predictive value; NPV, negative predictive value. Both OCB and intrathecal FLCK synthesis were specific for inflammatory neurological diseases (specificity 100%) while the sensitivity of FLCK (83%) exceeded the sensitivity of OCB (38%) and CSF-CC (66%) for the detection of inflammatory neurologic diseases.

## Discussion

In a cohort of 62 patients with a new onset (< 3 months) neuropsychiatric syndrome, we found only patients with retrospectively validated AIE and patients with infectious encephalitis to exhibit intrathecal FLCK synthesis at first admission. While the CSF white cell count tended to be higher in infectious encephalitis, the intrathecal fractions of FLCK did not discriminate between infectious encephalitis and AIE. However, intrathecal FLCK synthesis allowed us to stratify patients with neuropsychiatric syndromes into inflammatory vs. non-inflammatory etiologies. Here, intrathecal FLCK synthesis performed equally well as CSF pleocytosis. Moreover, in therapy-naive patients with neuropsychiatric syndromes that had a normal cell count in the CSF, elevated intrathecal FLCK indicated an inflammatory etiology (e.g. AIE^+^).

### Comparison of quantitative intrathecal Ig synthesis vs. OCB vs. FLCK synthesis

All patients with quantitative intrathecal Ig synthesis had CSF-specific OCB and showed intrathecal FLCK synthesis. Conversely, within OCB^+^ individuals, only a fraction also had quantitative intrathecal Ig synthesis, i.e. 50% in AIE^+^ (n = 3/6), 33% in AIE^–^ (n = 2/6), and 80% in INE (n = 4/5). An even smaller fraction of all FLCK-IF^+^ individuals showed intrathecal Ig synthesis, i.e. 20% in AIE^+^ (n = 3/15), 29% in AIE^–^ (n = 2/7), and 44% in INE (n = 4/9). The overall presence of OCB in our diagnostic groups—50% in INE, 33% in AIE^–^, 32% in AIE^+^, and none in DGN and NIND control individuals—was consistent with previous reports^17–19^. In particular in AIE, the presence of OCB varies substantially, with the highest incidences in anti-GABA(b) and anti-NMDA antibody-positive AIE and the lowest incidences in anti-LGI-1 and anti-IgLON5 antibody-positive AIE^17–19^. When comparing intrathecal FLCK synthesis with CSF-specific OCB, intrathecal FLCK synthesis detected inflammatory etiologies in our cohorts with equal specificity but higher sensitivity than OCB (Table 2), which is in line with a recent report^8^. Since none of the individuals in our NIND cohort exhibited intrathecal FLCK synthesis, intrathecal FLCK synthesis reached a specificity of 100% in identifying an inflammatory etiology. However, in larger cohorts, the increased sensitivity of intrathecal FLCK synthesis for the detection of inflammatory etiologies is associated with a decreased specificity as compared to OCB. For instance, in multiple sclerosis, the specificity of intrathecal FLCK synthesis at a threshold index of 5 was reported to be 90.4%, while the specificity of OCB reached 95.2%^20^. Notably, the negative predictive value of intrathecal FLCK synthesis, which largely exceeds that of OCB and CSF pleocytosis (Table 2), might help to rule out inflammatory etiologies in patients with subacute onset neuropsychiatric syndromes, in particular since the measurement of intrathecal FLCK synthesis was very robust while the determination of OCB is known to be variable according to disease phase, quality of the immune response (IgM, IgA, IgG), but also technical challenges^12,19^.

### Intrathecal FLCK as a tool to help clinical decision-making in patients with neuro-psychiatric syndromes

Inflammatory CSF transformation is widely considered as a key feature of AIE. Nevertheless, the degree of inflammatory CSF transformation can differ substantially across the subtypes of AIE. For instance, anti-NMDAR antibody encephalitis presents with CSF pleocytosis in about 80% of the cases, while anti-LGI-1 and anti-CASPR2 antibody positive encephalitis often exhibit normal CSF standard parameters^18,19^. Irrespective of the antibody subtype and the degree of inflammatory CSF alterations, all patients with AIE^+^ (after exclusion of paraneoplastic syndromes) had intrathecal FLCK synthesis at first admission, very similar to INE patients. This is consistent with previous studies reporting elevated levels of intrathecal FLCK in tick-borne encephalitis, Epstein-Barr-Virus-related encephalitis, Varicella-Zoster-Virus-related encephalitis, as well as bacterial encephalitis^21^.

In contrast to AIE^+^, only about 40% of AIE^–^ patients showed intrathecal FLCK synthesis. It is possible that pathogenic events other than autoantibody-mediated functional or structural perturbations (such as T cell-mediated autoimmune pathology) might be operational in AIE^–^ patients without intrathecal FLCK synthesis. However, due to the retrospective design of our study, many patients may also have been misclassified as AIE in their medical records. In fact, only 9 of the 18 individuals in the AIE^–^ group fulfilled the recently published criteria for AIE, which we applied retrospectively to our cohort based on medical records^3^. Seven of these 9 individuals (78%) presented with intrathecal FLCK synthesis, and 6 (67%) showed a response to first-line immunotherapy (corticosteroids or plasmapheresis) in terms of functional improvement or structural response in follow-up MRI imaging. Interestingly, the post-hoc categorization as probable or possible AIE by clinical criteria and the response to immunotherapy corresponded well with the presence of intrathecal FLCK synthesis (Supplementary File 2). As recently emphasized, misdiagnosis of AIE occurs commonly even in specialized centers and leads to morbidity from unnecessary immunotherapies or delayed treatment of the correct diagnosis, respectively. A leading cause of misdiagnosis has been shown to be the insufficient application of the diagnostic criteria, but also the variable spectrum of immunohistochemical and cell-based assays used throughout institutions. While narrow-spectrum assays fail to detect known antibodies, the detection of new antibodies is limited to research laboratories^22–24^. In fact, the steadily growing incidence of AIE in the last two decades is in part due to the discovery of novel anti-neural autoantibodies^2^. In this context, measurements of intrathecal FLCK synthesis could help to preselect candidates for extensive antibody testing in a notoriously heterogeneous clinical scenario of potential AIE patients.

When the CSF white cell count is normal in a population with suspected AIE, elevated intrathecal FLCK could identify those patients who later test positive for AIE-associated antibodies. In those cases without intrathecal FLCK synthesis, however, inflammatory etiologies are unlikely, directing the clinical work-up toward alternative etiologies. Of note, none of the patients with paraneoplastic syndromes in our cohort (anti-Yo, anti-Ma2, anti-SOX1, and anti-VGCC) showed intrathecal FLCK synthesis, even if the CSF white cell count was elevated. Patients with encephalopathies due to neurodegenerative disease (“symptomatic controls”; comprising dementia, prion disease, amyotrophic lateral sclerosis, tauopathies, and synucleinopathies) and non-inflammatory neurological disease controls exhibited no intrathecal FLCK synthesis. This argues for an accurate selection of our NIND controls. In fact, in other NIND cohorts, intrathecal FLCK synthesis was reported in about 6.5% of cases^8^.

While FLCK production rather reflects B-cell-mediated inflammatory events, other (more T cell/macrophage-mediated inflammatory processes) might not translate to similarly high concentrations of FLCK, which could explain variable findings of IF-FLCK in heterogenous inflammatory neurologic diseases. As recently published, cellular peripheral blood and CSF profiling improve the differential diagnosis of neuroinflammatory disorders^6^. Here, in the search for group-specific cell fraction patterns within the inflammatory groups (AIE^+^, AIE^–^, and INE), only the B cell fraction differed substantially between AIE^+^ and INE, likely indicating diverse cellular response patterns at symptom onset in viral and autoimmune encephalitis. Notably, IF-FLCK correlated moderately with the intrathecal B cell/plasma blast fractions and with the CSF white cell count.

Based on our findings in this cohort of suspected AIE cases, the presence of intrathecal FLCK synthesis should raise suspicion of an inflammatory etiology, favoring autoimmune over infectious encephalitis in the absence of CSF pleocytosis. In the presence of CSF pleocytosis, intrathecal FLCK has no added value to the diagnosis of AIE. In the absence of both CSF pleocytosis and intrathecal FLCK synthesis, non-inflammatory etiologies should be considered. Thus, measuring intrathecal FLCK may prevent unnecessary antibody testing, and conversely, when AIE is suspected, elevated IF-FLCK but normal CSF cell counts should encourage a continued search for autoantibodies.

### Confounding factors of intrathecal FLCK synthesis

Intrathecal FLCK synthesis has been considered a methodologically robust parameter. In the present study, we used the hyperbolic reference range for Q_FLCK_, which is well accepted in German-speaking countries, whereas previous international studies relied on the linear FLCK index or exponential curves^8,14,15,19^. More importantly, potential pre-analytic confounders of the determination of intrathecal FLCK synthesis are immunomodulatory or immunosuppressive therapies and renal function and must be considered when interpreting FLCK measurements in CSF and serum.

Corticosteroid therapy decreases FLCK concentrations in the serum^9^. Here, we excluded all individuals on steroids or other immunomodulatory agents from the analysis. This strategy might explain some differences in the fraction of intrathecal FLCK synthesis in our patients with inflammatory etiologies as compared to the fraction of intrathecal FLCK synthesis in inflammatory CNS diseases reported by other investigators^8^.

The likelihood of intrathecal FLCK synthesis decreases with declining renal function due to an increase in serum concentrations of FLCK. Even though Reiber's method is more resilient to the influence of impaired renal function than the linear FLCK index, renal function has to be taken into consideration. We did not observe an unbalanced distribution of individuals with renal failure in our diagnostic groups (one AIE^+^, two AIE^-^, and one NIND). Therefore, we do not consider our interpretation to be substantially biased due to renal function impairment, in particular since this would rather produce a false negative than a false positive classification of intrathecal FLCK synthesis.

### Limitations of the study

First, the post-hoc assignment of patients to the diagnostic groups based on medical records might have introduced a bias. As mentioned above, the retrospective application of the published AIE diagnostic criteria^3^ on the AIE^–^ group led us to reconsider the diagnosis in half of the individuals in the AIE^–^ group based on the published diagnostic criteria. Although this links our study to the routine of clinical practice—since all enrolled individuals (except the controls) shared the initial clinical suspicion of AIE—it also stresses the need to strictly adhere to the diagnostic criteria. Besides the retrospective design, further limitations of our study are due to the small group sizes and single measurements of FLCK in blood and CSF due to the limited availability of material. Even though our study was adequately powered to detect large differences between the groups and the results were significant, our findings will need to be replicated in larger-scale multicenter approaches before any implementation into clinical practice can be considered.

### Concluding remarks

In the setting of a new onset neuropsychiatric syndrome and otherwise missing inflammatory CSF transformation, the intrathecal synthesis of FLCK may predict an inflammatory etiology such as antibody-positive autoimmune encephalitis with high sensitivity and specificity. Conversely, AIE^+^ appears unlikely in the event of both normal CSF standard parameters and the absence of intrathecal FLCK synthesis. We propose that intrathecal FLCK may be exploited as a predictor of possible AIE in patients with neuropsychiatric syndromes without CSF pleocytosis. Larger cross-sectional studies are required to confirm this hypothesis.

## Methods

### Patients and ethics statement

We retrospectively included patients with first-episode subacute-onset neuropsychiatric syndromes undergoing diagnostic work-up for AIE at the Department of Neurology of the Technical University of Munich School of Medicine between 2015 and 2020. This study was approved by the ethics committee of the Technical University of Munich School of Medicine (approval number 94/21 S-EB). All methods were carried out in accordance with relevant guidelines and regulations. Informed consent was obtained from all study subjects or their legal guardians.

As part of routine diagnostics at our department, freshly drawn serum and CSF samples were frozen within 30 min and stored at − 80 °C in our biobank. Flow cytometric analysis of immune cell subsets (FACS) was routinely performed on fresh CSF samples. Paired blood and CSF samples dating to the first admission were taken from our biobank. Course of disease and discharge diagnoses were extracted from medical records.

Patients with insufficient clinical data or incomplete biosamples were excluded from the evaluation, as were patients on immunosuppressive therapies. We also excluded cases with metabolic, toxic, neoplastic, and genetic etiologies of neuropsychiatric syndromes as well as delirium. Of note, cases with retrospectively assigned autochthonous psychiatric diagnoses as a large group of differential diagnoses were also excluded from the evaluation.

Eventually, our cohort was defined as a cohort of patients with an encephalopathic syndrome suggestive of or compatible with AIE, characterized by rapid progression of fewer than 3 months, working memory deficits, changes in behavior or mental status (altered level of consciousness, lethargy, or personality change), or psychiatric symptoms. All enrolled patients (except patients from the control group) were tested for CSF autoantibodies, including anti-Hu, anti-Yo, anti-Ri, anti-ANNA-3, anti-Tr/DNER, anti-myelin, anti-Ma/Ta, anti-GAD65, anti-amphiphysin, anti-aquaporin-4 (AQP4), anti-glutamate receptors (type NMDA, AMPA), anti-GABA_A/B_ receptors, anti-LGI-1, anti-CASPR2, anti-ZIC4, anti-DPPX, anti-glycin receptors, anti-AGNA, anti-mGluR1, anti-mGluR5, anti-Rho-GTPase activating protein 26, anti-ITPR1, anti-Homer 3, anti-myelin oligodendrocyte glycoprotein (MOG), anti-neurochondrin, anti-GluRD2, anti-flotillin 1/2, anti-IgLON5, anti-CV2, anti-PNMA2, anti-VGCC, anti-recoverin, and anti-SOX1. The cohort was then stratified according to the following criteria:

AIE^+^—detectable autoantibody (cell-surface or intracellular antigens) in the CSF and absence of an infectious agent; AIE^–^—absence of autoantibodies and infectious agents in the CSF, also not meeting diagnostic criteria for DGE; INE—absence of autoantibodies and detection of an infectious agent in the CSF by PCR; DGE—patients with a syndrome fulfilling diagnostic criteria for neurodegenerative diseases^25–31^ without CSF autoantibodies. The control group, NIND (non-inflammatory neurological disease), was defined by the consensus definitions for control groups in CSF biomarker studies^32^. After pseudonymization, the investigators were blinded to the diagnoses until the end of the analysis.

### Laboratory analysis

Paired CSF and serum samples stored at – 80 °C were analyzed for FLCK levels using the N Latex FLC kappa kit (Siemens Healthcare Diagnostics Products GmbH, Marburg, Germany) according to the manufacturer’s protocol on a Siemens BN Prospec nephelometer. CSF predilution was set to 1:1; serum predilution was set to 1:100. According to the manufacturer, the lower limit of detection was 0.034 mg/L. The hyperbolic reference range and the intrathecal fraction of FLCK (IF-FLCK) were calculated according to the formula by Reiber et al. (K_IF_ = (1 − Q_lim_/Q_Kappa_) × 100 [%]) with Q_Kappa_ as the CSF/serum quotient of FLCK using the CSF-Tool software from Albaum IT solutions (W. Albaum, FLCK Statistics and Graphic Program, Freeware www.albaum.it 7 (2019))^14^. Immunoglobulins and albumin were measured by immunonephelometry on the same Siemens BN Prospec nephelometer according to the manufacturer’s protocol. Quantitative expressions of the intrathecal humoral immune response were based on the calculation of the CSF/serum ratios of IgG, IgM, and IgA with Q_Ig_ = Ig_CSF_ [mg/L]/Ig_serum_ [g/L]. The upper limits of the respective reference ranges, Q_lim_(IgG), Q_lim_(IgM), and Q_lim_(IgA), were calculated against Q_Alb_ according to Reiber’s revised hyperbolic function. Values for Q (Ig) exceeding Q_lim_ (Ig) were considered to indicate an intrathecal immunoglobulin synthesis^33^. The CSF/serum albumin ratio, Q_Alb_ = Alb_CSF_ [mg/L]/Alb_serum_ [g/L], was used to assess blood-CSF-barrier function. Blood-CSF-barrier dysfunction was defined as Q_Alb_ > 10 × 10^–3^ for an age of 60 years or older, Q_Alb_ > 8 × 10^–3^ for an age of 40–59 years, and Q_Alb_ > 6.5 × 10^–3^ for an age of 39 years or younger. Oligoclonal Ig bands (OCB) were assessed by isoelectric focussing and consecutive silver staining in polyacrylamide gels as described previously^34^. Following the European consensus standards on CSF analysis in Multiple Sclerosis (MS), we classified positive OCB as pattern type 2 or type 3^35^.

Antibody testing was performed using cell-based assays (CBAs) and consequently confirmed using immunofluorescence in commercial test kit panels (Euroimmun, Lübeck).

CSF white cell counts > 4/µL were classified as pleocytosis. FACS (Fluorescence activated cell sorting) was performed at the time of sampling as described previously^36^. Briefly, fresh CSF was immediately spun down (300 g for 10 min), the supernatant removed, and the pellet resuspended in phosphate-buffered saline (PBS) (PAA, Pasching, Austria) with 2% fetal calf serum (FCS) (Invitrogen, Darmstadt, Germany). After incubating with an antibody mix (20 min at 4 °C), cells were spun down, washed, and resuspended in PBS wash solution (including 2% FCS) for flow cytometric analysis (Beckman Coulter Cyan, Brea, CA, USA). The following antibodies were used for staining: CD4 PerCP, CD3 APC-Cy7, CD45 VM (all BD Bioscience, Bedford, MA, USA), CD19 ECD, CD56 APC, CD14 FITC, and CD138 PE (all Beckman Coulter). This allowed differentiating between CD4^+^ T cells (CD45^+^CD3^+^CD4^+^), CD8^+^ T cells (CD45^+^CD3^+^CD8^+^), monocytes (CD45^+^CD14^+^), NK cells (CD45^+^CD56^+^), B cells (CD45^+^CD19^+^CD138^−^), and plasma blasts (CD45^+^CD19^+^CD138^+^).

Renal function was assessed as part of routine diagnostics for all patients under investigation at the time of sampling and estimated by eGFR (mL/min/1.73 m^2^) according to the CKD-EPI equation^37^.

### Statistical analysis and data processing

Statistical analysis was performed using GraphPad Prism (La Jolla, CA, USA; version 8) and Python 3.10 (Python Software Foundation, https://www.python.org/). To compare two or more groups, the Mann–Whitney-U test or the Kruskal–Wallis test was used. The locally synthesized absolute amount of FLCK and IF-FLCK were calculated with respect to the Q_mean_ (value of the mean curve for the Q_Alb_ of the individual patient) for statistical group comparison. For ROC (Receiver Operating Characteristics)-analysis, the IF-FLCK was calculated in relation to Q_lim_ (upper limit of the reference range for the Q_Alb_ of the individual patient).

### Ethical approval and consent to participate

This study was approved by the ethics committee of the Technical University of Munich (approval number 94/21 S-EB).

### Supplementary Information

Supplementary Information 1.

Supplementary Information 2.

Supplementary Information 3.

Supplementary Information 4.

## Data Availability

Anonymized raw data will be made available upon appropriate request by qualified researchers. Please contact T. Korn (thomas.korn@tum.de).

## Abbreviations

AIE^+^: Antibody-positive autoimmune encephalitis
AIE^–^: Antibody-negative autoimmune encephalitis
INE: Infectious encephalitis (viral)
DGE: Encephalopathy due to degenerative disorders
NIND: Non-inflammatory neurological disease
CSF: Cerebrospinal fluid
FLCK: Free light chains kappa
IF-FLCK: Intrathecal fraction of FLCK
OCB: Oligoclonal bands
ROC: Receiver operating characteristics
AUC: Area under the curve
FACS: Fluorescence-activated cell sorting
PCR: Polymerase chain reaction
