# Supplementary material for: The role of intrathecal free light chains kappa for the detection of autoimmune encephalitis in subacute onset neuropsychiatric syndromes
Source: Sci Rep. 2023 Oct 11;13:17224. doi: 10.1038/s41598-023-44427-6 (PMC10567819; doi:10.1038/s41598-023-44427-6)
Supplement: Supplementary file 1 — Supplementary Information 1. [file 41598_2023_44427_MOESM1_ESM.pdf]

Supplementary File 1. AIE$^+$ characteristics

AIE+ Pat. Nr.	Subacute onset (rapid progression of less than 3 months) of working memory deficits (short-term memory loss), altered mental status, or psychiatric symptoms	New focal CNS findings	Seizures not explained by a previously known seizure disorder	CSF pleocytosis (white blood cell count of more than five cells per mm^3)	EEG with epileptic or slow-wave activity involving the temporal lobes	MRI features suggestive of encephalitis	Reasonable exclusion of alternative causes	Bilateral brain abnormalities on T2-weighted fluid-attenuated inversion recovery MRI highly restricted to the medial temporal lobes	Response to first-line immunotherapy	Intrathecal FLCK synthesis	Antibody specification
1	+	+	-	+	+	+	+	-	+	+	LGI-1
2	+	-	+	-	+	+	+	-	-	+	LGI-1
3	+	-	+	+	-	+	+	-	+	+	NMDA
4	+	+	-	+	-	-	+	-	+	+	IgLON5
5	+	+	-	+	-	+	+	-	n	-	AGNA
6	+	-	+	+	-	-	+	-	+	+	NDMA
7	+	-	-	+	+	+	+	-	+	+	GABA(a)
8	+	+	-	+	+	+	+	-	+	+	NMDA
9	+	-	-	+	-	+	+	-	+	+	NMDA
10	+	+	+	+	-	-	+	-	+	-	Anti-Yo
11	+	+	-	-	n	-	+	-	-	-	Ma2
12	+	+	-	-	-	-	+	-	+	+	CASPR2
13	+	+	-	-	+	-	+	-	-	-	VGCC
14	+	-	-	-	-	+	+	-	+	+	LGI-1
15	+	+	-	-	n	-	+	-	-	+	GAD65
16	+	-	+	-	-	+	+	-	+	+	NMDA
17	+	+	+	-	-	+	+	-	+	+	NMDA
18	+	+	+	+	+	+	+	+	+	+	LGI-1
19	+	+	-	-	-	+	+	-	+	+	DPPX

Supplementary File 1. Clinical and paraclinical characteristics of the antibody-positive AIE (AIE$^+$) group. All 19 patients of the AIE+ group fulfilled the previously defined diagnostic criteria for at least "possible AIE" on admission and tested positive for intrathecal antibodies during the diagnostic work-up, allowing for the diagnosis of “definite” AIE. These criteria were retrospectively applied in our cohort of suspected AIE cases (“+” applicable; “-“ not applicable; “n” not applied).
