# Supplementary material for: The role of intrathecal free light chains kappa for the detection of autoimmune encephalitis in subacute onset neuropsychiatric syndromes
Source: Sci Rep. 2023 Oct 11;13:17224. doi: 10.1038/s41598-023-44427-6 (PMC10567819; doi:10.1038/s41598-023-44427-6)
Supplement: Supplementary file 2 — Supplementary Information 2. [file 41598_2023_44427_MOESM2_ESM.pdf]

Supplementary File 2. AIE^{-} characteristics

AIE^{-} Pat. Nr.	Subacute onset (rapid progression of less than 3 months) of working memory deficits (short-term memory loss), altered mental status, or psychiatric symptoms	New focal CNS findings	Seizures not explained by a previously known seizure disorder	CSF pleocytosis (white blood cell count of more than five cells per mm^3)	EEG with epileptic or slow-wave activity involving the temporal lobes	MRI features suggestive of encephalitis	Reasonable exclusion of alternative causes	Bilateral brain abnormalities on T2-weighted fluid-attenuated inversion recovery MRI highly restricted to the medial temporal lobes	Response to first-line immuno-therapy	Intrathecal FLCK synthesis
1	+	+	-	+	n	+	-	-	-	-
2	+	+	-	-	-	-	+	-	-	-
3	+	+	-	+	n	+	+	-	+	+
4	+	+	-	+	-	-	+	-	+	+
5	-	+	+	+	+	+	-	-	n	-
6	+	+	-	+	n	+	+	-	+	+
7	+	+	+	-	+	+	+	-	+	+
8	+	+	-	+	-	-	+	-	n	+
9	+	-	-	+	-	-	+	-	+	+
10	+	+	-	+	-	+	+	-	n	-
11	+	+	-	-	+	-	-	-	n	-
12	+	+	-	-	-	+	-	-	-	-
13	+	-	-	-	-	+	+	-	+	+
14	-	-	+	-	n	+	-	-	n	-
15	-	+	-	-	-	-	-	-	n	-
16	+	+	-	-	-	+	-	-	+	-
17	+	+	-	-	n	-	-	-	+	-
18	+	-	-	-	-	+	-	-	-	-

Supplementary File 2. Characteristics of the antibody-negative (AIE^{-}) group. Of 18 patients labeled as potential AIE on admission, 9 fulfilled the previously defined diagnostic criteria for at least "possible AIE". These criteria were retrospectively applied in our cohort of suspected AIE cases (“+” applicable; “-“ not applicable; “n” not applied).
