# Supplementary material for: The role of intrathecal free light chains kappa for the detection of autoimmune encephalitis in subacute onset neuropsychiatric syndromes
Source: Sci Rep. 2023 Oct 11;13:17224. doi: 10.1038/s41598-023-44427-6 (PMC10567819; doi:10.1038/s41598-023-44427-6)
Supplement: Supplementary file 3 — Supplementary Information 3. [file 41598_2023_44427_MOESM3_ESM.pdf]

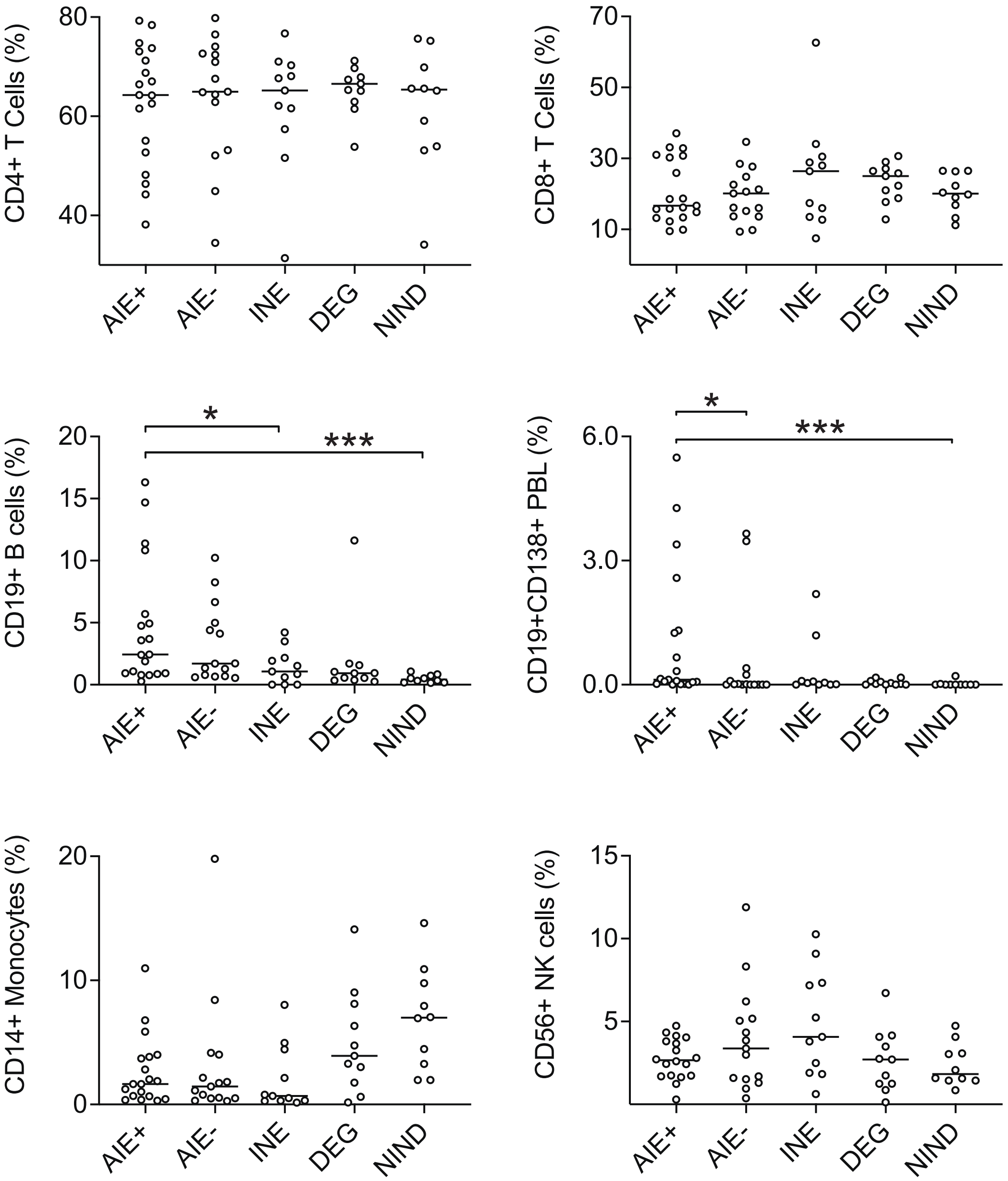

Supplementary File 3. Group-specific CSF immune cell fractions. $CD4^{+}$ and $CD8^{+}$ T cells, $CD19^{+}$ B cells, $CD19^{+}CD138^{+}$ immature plasma blasts (PBL), $CD14^{+}$ monocytes, and $CD56^{+}$ NK cells. (* p ≤ 0.05, ** p ≤ 0.01, *** p ≤ 0.001, **** p ≤ 0.0001).
