# Supplementary material for: The role of intrathecal free light chains kappa for the detection of autoimmune encephalitis in subacute onset neuropsychiatric syndromes
Source: Sci Rep. 2023 Oct 11;13:17224. doi: 10.1038/s41598-023-44427-6 (PMC10567819; doi:10.1038/s41598-023-44427-6)
Supplement: Supplementary file 4 — Supplementary Information 4. [file 41598_2023_44427_MOESM4_ESM.pdf]

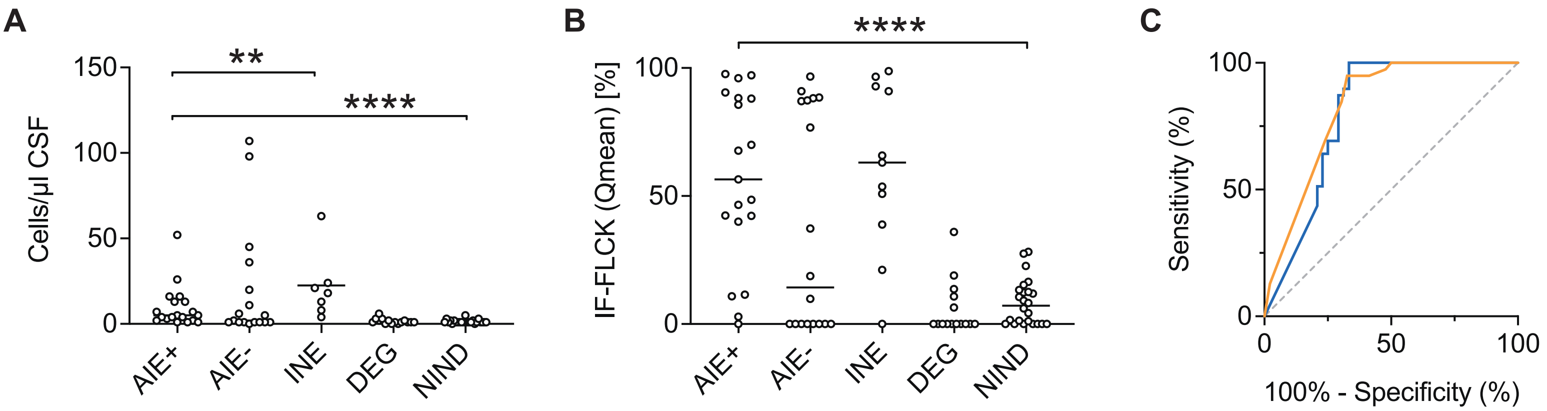

Supplementary File 4. Comparison of IF-FLCK and CSF white cell count. (A) Group-specific CSF white cell counts. **(B)** Group specific IF-FLCK, IF-FLCK (Qmean). **(C)** Receiver operating characteristics for IF-FLCK > Qlim (blue line, AUC = 0.809, p = 0.001) and CSF white cell count (orange line, AUC = 0.852, p = 0.0002) as to the differentiation of inflammatory (AIE^+, AIE^-, INE) vs non-inflammatory (DGE) encephalopathy and NIND. (* p ≤ 0.05, ** p ≤ 0.01, *** p ≤ 0.001, **** p ≤ 0.0001).
